# MicroRNA-132-3p, Downregulated in Myeloid Angiogenic Cells from Hereditary Hemorrhagic Telangiectasia Patients, Is Enriched in the TGFβ and PI3K/AKT Signalling Pathways

**DOI:** 10.3390/genes13040665

**Published:** 2022-04-09

**Authors:** Anthony Cannavicci, Qiuwang Zhang, Marie E. Faughnan, Michael J. B. Kutryk

**Affiliations:** 1 Institute of Medical Science, Cardiovascular Sciences Specialization Program, University of Toronto, Toronto, ON M5S 1A8, Canada; a.cannavicci@mail.utoronto.ca (A.C.); marie.faughnan@unityhealth.to (M.E.F.); 2 Keenan Research Center for Biomedical Science, Division of Cardiology, St. Michael’s Hospital, Unity Health Toronto, Toronto, ON M5B 1T8, Canada; qiuwang.zhang@unityhealth.to; 3 Toronto HHT Centre, Li Ka Shing Knowledge Institute, St. Michael’s Hospital, Unity Health Toronto, Toronto, ON M5B 1T8, Canada; 4 Department of Medicine, Division of Respirology, University of Toronto, Toronto, ON M5S 3H2, Canada

**Keywords:** hereditary hemorrhagic telangiectasia, myeloid angiogenic cells, microRNAs, early endothelial progenitor cells, circulating angiogenic cells, transforming growth factor beta signalling pathway, PI3K/AKT signalling pathway

## Abstract

Background. Hereditary hemorrhagic telangiectasia (HHT) is a rare, autosomal dominant genetic disorder characterized by life-threatening vascular dysplasia. Myeloid angiogenic cells (MACs), alternatively called early endothelial progenitor cells or circulating angiogenic cells, do not directly incorporate into developing blood vessels, but augment angiogenesis in a paracrine manner. MAC dysfunction has been reported in HHT. MicroRNAs (miRNAs) regulate cellular function by modulating gene expression post-transcriptionally. To date, the role of miRNAs in HHT MAC dysfunction has not been documented. Objective. The goal of this study was to comparatively profile miRNAs in HHT patient and control MACs to identify dysregulated miRNAs that may be responsible for the observed MAC dysfunction in HHT. Methodology/Results. Twenty-three dysregulated miRNAs (twenty-one upregulated and two downregulated) in HHT MACs were identified with a TaqMan miRNA microarray. Pathway enrichment analysis showed that the dysregulated miRNAs were significantly enriched in pathways involved in HHT pathogenesis, such as the transforming growth factor β (TGFβ), phosphatidylinositol 3-kinase/protein kinase B (PI3K/AKT), and Hippo signalling pathways. Furthermore, miR-132-3p was determined to be significantly reduced in HHT MACs compared with controls by reverse transcription-quantitative polymerase chain reaction (RT-qPCR). Bioinformatic analysis revealed that miR-132-3p is significantly enriched in the TGFβ and PI3K/AKT signalling pathways, targeting *SMAD4*, an effector of the TGFβ signalling pathway and *RASA1*, a negative regulator of the PI3K/AKT signalling pathway, respectively. Conclusion. MiRNA dysregulation, specifically reduced expression of miR-132-3p, in HHT MACs was identified. The dysregulated miRNAs are significantly enriched in the TGFβ, PI3K/AKT, and Hippo signalling pathways. These data suggest that alteration in miRNA expression may impair these pathways and contribute to MAC dysfunction in HHT.

## 1. Introduction

Hereditary hemorrhagic telangiectasia (HHT) is a rare, autosomal dominant, genetic disorder characterized by life-threatening vascular malformations. Approximately 1 in 5000 to 8000 people are affected globally [1]. Patients can develop skin and mucocutaneous vascular malformations, that are direct connections between arterioles and venules lacking capillaries, called telangiectases [2]. Approximately 95% of patients develop recurrent and spontaneous epistaxis from nasal telangiectases [3]. Patients can also develop direct connections between arteries and veins, lacking capillaries, in the lungs, brain, liver, and spine, called arteriovenous malformations (AVMs) [4]. Serious complications can arise from AVMs, including high output cardiac heart failure, cirrhosis, ischemic and hemorrhagic stroke, and brain abscess [5]. HHT is underdiagnosed and lacks an effective pharmacological therapy.

Heterozygous mutations in at least three genes, including endoglin (*ENG*, chromosomal locus 9q34), activin-A type-II receptor-like kinase 1 (*ACVRL1*, also known as *ALK1*, chromosomal locus 12q1), and mothers against decapentaplegic homolog 4 (*SMAD4*, chromosomal locus 18q21) are known to cause HHT [6,7,8]. Mutations in *ENG* and *ACVRL1* lead to HHT Type 1 and 2, respectively, while *SMAD4* mutations result in a combined juvenile polyposis-HHT syndrome (JP/HHT). HHT Type 1 and 2 comprise 70–90% of cases, while JP/HHT is responsible for approximately 1–2% [9,10,11]. Over 850 pathogenic mutations in *ENG*, *ACVRL1*, and *SMAD4* have been documented (https://arup.utah.edu/database/HHT/, https://arup.utah.edu/database/SMAD4/SMAD4_welcome.php, accessed on 24 January 2022). These genes play critical roles in the transforming growth factor β/bone morphogenetic protein (TGFβ/BMP) signalling pathway. This pathway regulates vascular homeostasis, endothelial cell function, and angiogenesis [12]. *ENG* encodes a TGFβ co-receptor or auxiliary receptor that is responsible for maintaining a high affinity bond between TGFβ ligands and receptors. *ACVRL1* encodes a TGFβ receptor I that binds to TGFβ ligands with *ENG* and TGFβ receptor II. SMAD4 is an intracellular effector of TGFβ/BMP signalling that upon activation translocates to the nucleus to regulate gene expression. Mouse models of HHT were integral in the characterization of the pathogenicity of these mutations, as well as the identification of endothelial cells (ECs) as the predominant pathologic cell [13]. HHT animal models have also led to the development of the “Three Event Hypothesis” that states AVM formation is due to the synergy of three events: the inherited mutation, loss of heterozygosity of the inherited mutation, and an angiogenic trigger [13]. Indeed, Snellings et al. demonstrated that HHT Type 1 and 2 telangiectasia biopsies could have bi-allelic loss of *ENG* or *ACVRL1* [14]. This suggests that the genetic mutations alone are necessary, but not sufficient to generate vascular malformations, and alternative biological factors must be at play.

MicroRNAs (miRNAs) are short (21–25 nucleotides long) non-coding RNA molecules that regulate gene expression in a post-transcriptional fashion [15]. To date, over 2000 miRNAs have been discovered and are involved in virtually every cellular process [16,17,18]. MiRNAs regulate gene expression by targeting the 3′ untranslated region of messenger RNA (mRNA), where perfect complementarity results in mRNA cleavage and imperfect complementarity results in mRNA silencing through the blockage of translational machinery [16,19]. The latter mechanism is typically carried out by human miRNAs [19]. These promiscuous molecules have been shown to possess tens to hundreds of targets and regulate approximately 30% of known genes [20,21]. MiRNAs are involved in a variety of human diseases and have been established as reliable biomarkers, especially in oncology [22,23]. We have previously reported reduced levels of miRs-28-5p and -361-3p in HHT patient peripheral blood mononuclear cells (PBMCs), as well as elevated levels of circulating miR-210 in HHT patients with pulmonary AVMs [24,25]. Various other studies have identified miRNA dysregulation in HHT, but miRNA research in HHT is limited and an exact pathogenic role of any one miRNA has yet to be fully elucidated [26,27,28].

Myeloid angiogenic cells (MACs) [29], also known as early endothelial progenitor cells [30,31,32] or circulating angiogenic cells [33], are myeloid cells of the hematopoietic lineage with potent pro-angiogenic and vasoreparative properties [34,35]. They are derived through the culture of PBMCs on fibronectin-coated flasks in vascular endothelial growth factor (VEGF)-containing medium for 4–7 days [36,37,38]. MACs do not directly incorporate into a developing blood vessel, but rather support angiogenesis in a paracrine manner through the secretion of various growth factors, including VEGF, interleukin 8 (IL8), stromal cell-derived factor 1 (SDF1), insulin-like growth factor 1 (IGF1), and hepatocyte growth factor (HGF) [39,40].

MAC dysfunction has been shown in a variety of human diseases [35,36,37,38,41,42]. van Laake et al. demonstrated that MACs from HHT Type 1 patients have impaired migration and homing to sites of cardiac injury in a mouse model of myocardial infarction [41]. Tepper et al. showed that MACs derived from type 2 diabetes patients had reduced proliferation, decreased adherence to human umbilical vein endothelial cells (HUVECs), and a decreased ability to augment tube formation [36]. Vasa et al. showed that patients with coronary artery disease (CAD) had reduced MAC levels that were correlated with CAD risk factors, such as smoking and diabetes [37]. They also demonstrated that CAD MACs had reduced migratory capacity [37]. Ward et al. demonstrated that MACs from patients with CAD had impaired vasoreparative properties, blunted migration, and reduced expression of VEGF and platelet derived growth factor [38]. It was also found that endothelial nitric oxide synthase (eNOS) overexpression in CAD MACs improved HUVEC tube formation and augmented neovascularization and perfusion in a nude mouse model of hind limb ischemia [38]. Zhang et al. identified reduced migration and increased apoptosis in MACs derived from patients with idiopathic pulmonary arterial hypertension [43]. Most relevant to the present study, Zucco et al. demonstrated that MACs derived from HHT patients had impaired function [44]. The exact mechanism of MAC dysfunction in HHT is not completely understood and whether miRNA dysregulation is involved has not yet been investigated. In this study, we sought to comparatively profile miRNAs in HHT patient and control MACs to identify dysregulated miRNAs that may play a role in HHT MAC dysfunction.

## 2. Methodology

### 2.1. Patient Recruitment and Ethics Statement

Informed written consent was obtained from all study participants, i.e., HHT patients and age- and gender-matched controls. Forty HHT patients between the ages of 18 and 65 who were clinically diagnosed with HHT according to the Curaçao diagnostic criteria for HHT were recruited [45]. HHT patients who demonstrated clinically significant anemia (hemoglobin < 100 g/L) or pregnancy were excluded to prevent risk of health complications, e.g., worsening of anemia that may require blood transfusion. All HHT patients were recruited from the Toronto HHT Centre at St. Michael’s Hospital, Toronto, Ontario, Canada. The study protocol was approved by the Research Ethics Board of St. Michael’s Hospital, University of Toronto (REB 02-185), in accordance with the Code of Ethics of the World Medical Association (Declaration of Helsinki).

### 2.2. MAC Cell Culture

Vacutainer CPT Mononuclear Cell Preparation Tubes (BD Biosciences, Mississauga, ON, Canada) containing Ficoll-Hypaque solution were used for PBMC isolation. A total of 24 mL of peripheral blood was obtained in 3 tubes. After centrifugation at room temperature at 1650× *g* for 30 min, PBMCs were carefully collected from the buffy coat, washed one time with phosphate buffered saline (PBS), and seeded at a density of 0.75 × 10^6^ cells/cm^2^ on human fibronectin-coated (10 μg/mL) T25 flasks in complete Endothelial Cell Growth Medium-2 (EGM-2) (Lonza, EGM-2 BulletKit, cat# CC-3162) supplemented with 20% human serum. On the 3rd day of culture, non-adherent cells were removed through aspiration and fresh growth medium was supplied. Media were replenished every other day until day 7 when cells were used directly for analysis. MACs were analyzed by cell uptake of Dil-Ac-LDL, binding of UEA-Lectin, and detection of VEGFR2 expression, as previously described [38,44,46,47].

### 2.3. Total RNA Isolation from MACs

A Qiagen RNeasy Mini Kit (Qiagen, Toronto, ON, Canada; cat# 217004) was used to isolate total RNA from MACs in accordance with the manufacturer’s instructions. Briefly, cells were lysed with 700 μL of Qiazol lysis reagent and the lysate was incubated for 5 min at room temperature and mixed with 140 μL of chloroform. After the mixture was centrifuged at 12,000× *g* for 15 min at 4 °C, 300 μL of the aqueous layer containing RNA was carefully extracted and mixed with 450 μL of 100% ethanol. Subsequently, 700 μL of this mixture was added to an RNeasy Mini column and centrifuged at 10,000× *g* for 15 s at 4 °C. Then the column was washed twice with 500 μL of Buffer RPE followed by 500 μL of 100% ethanol at 10,000× *g* for 15 s at 4 °C. Finally, RNA was eluted with 30 μL of RNase free water at 10,000× *g* for 1 min at 4 °C. A NanoDrop 2000 spectrophotometer was used to assess the concentration and quality of RNA prior to storage at −80 °C. All components used were free of DNase, RNase, and pyrogen.

### 2.4. MiRNA Microarray

A TaqMan Low Density MicroRNA Microarray covering 377 human miRNAs (Applied Biosystems, Burlington, ON, Canada, Card A v2.0, cat# 4398965) was employed to profile miRNAs in 6 independent HHT and 6 independent control MAC RNA samples. Reverse transcription (RT) and miRNA array were performed as described elsewhere [48]. RQ Study software (Applied Biosystems) was used to analyze the array results and normalized to U6 snRNA as determined by the NormFinder Excel plugin (https://moma.dk/normfinder-software) (accessed on 18 March 2020) [49]. Select miRNAs of interest were determined based on a fold change of 1.5 or greater and <31 cycle threshold value (Ct) [50,51]. Relative quantification of the expression of individual mRNAs was carried out with the Livak method ($2^{-\Delta \Delta C_{t}}$) and normalized against endogenous U6 snRNA.

### 2.5. Enrichment Analysis of Dysregulated MiRNAs

DIANA-miRPath v.3 [52] (http://snf-515788.vm.okeanos.grnet.gr/) (accessed on 11 May 2020) was used to perform enrichment analysis on the 23 dysregulated miRNAs identified by microarray analysis after systematic exclusion. The microT-CDS prediction algorithm with a threshold of 0.5 was applied to identify targets of the 23 dysregulated miRNAs followed by a functional enrichment analysis on the identified targets with the Kyoto Encyclopedia of Genes and Genomes (KEGG) pathways annotation database. Statistical analysis was performed with Fisher’s exact test with a *p*-value threshold of 0.01 and corrected with the false discovery rate (FDR). Results were combined at the pathways level (pathways union) and presented as a heat map with hierarchical clustering. MiR-886-5p was excluded from the analysis by DIANA-miRPath v.3 because it had been withdrawn from miRBase (v22.1) due to a lack of empirical evidence to support its existence.

### 2.6. RT-qPCR for MiRNA Validation

Selected dysregulated miRNAs identified by the miRNA microarray analysis were validated with RT-qPCR. The RT reaction was performed with a total volume of 15 μL and was comprised of: 3 μL 5X RT primer, 7 μL RT master mix, and 5 μL RNA sample (20 ng of total RNA). For each RT reaction, the master mix was prepared as follows: 0.15 μL 100 mM dNTP, 0.19 μL (20 U/μL) RNase inhibitor, 1 μL (50 U/μL) MultiScribe Reverse Transcriptase, 1.5 μL 10X reverse transcription buffer, and 4.16 μL of nuclease-free water. A Veriti 96-well thermal cycler was used to perform RT according to the following protocol: 16 °C for 30 min, 42 °C for 30 min, 85 °C for 5 min, and 4 °C hold. Quantitative PCR was performed in a total volume of 10 μL consisting of: 0.5 μL 20X miRNA PCR primer, 1 μL RT product, 5 μL 2XTaqMan Fast Advanced Master Mix, and 3.5 μL of nuclease-free water. A QuantStudio 7 Flex Real-Time PCR System (Applied Biosystems) was used to perform qPCR according to the following thermocycling protocol: 95 °C for 20 s, and 40 cycles of 95 °C for 1 s and 60 °C for 20 s. Relative quantification of miRNAs normalized against endogenous U6 snRNA was performed using the Livak method ($2^{-\Delta \Delta C_{t}}$).

### 2.7. Enrichment Analysis of MiR-132-3p

MIENTURNET [53] (http://userver.bio.uniroma1.it/apps/mienturnet/) (accessed on 19 November 2021) and DIANA-miRPath v.3 were used to identify miR-132-3p targets and subsequently perform functional enrichment analysis. DIANA-miRPath v.3 parameters and methodology were described above. MIENTURNET utilized the experimentally validated miR–target interactions database, miRTarbase [54] (Release 7.0, September 2017), to identify targets of miR-132-3p. A threshold of 1 was used for the minimum number of miR–target interactions and for the FDR adjusted *p*-value. Targets were filtered for strong empirical evidence such as Western blot and luciferase assay. Functional enrichment was performed on these identified targets using the Reactome pathways database. MiR-886-5p was excluded from the analysis as described above.

### 2.8. Statistical Analyses

Data were presented as the mean ± standard deviation (SD). An unpaired two-tailed Student’s *t*-test with Welch’s correction was used for all statistical analyses. A *p*-value < 0.05 was considered statistically significant. Z-score analysis was performed with a threshold of 2 SDs for outlier identification.

## 3. Results

### 3.1. Study Participants

A total of 40 HHT patients (21 females and 19 males) and 22 controls (12 females and 10 males) were recruited to participate in this study (Table 1). The mean age of the HHT patient and control groups were 49.4 ± 10.6 years and 46.2 ± 12.6 years, respectively. Pulmonary AVMs were detected by thoracic computed tomography (CT) or pulmonary angiography, cerebral AVMs were detected by magnetic resonance imaging (MRI), and symptomatic hepatic or liver vascular malformations (VMs) were detected by MRI, contrast-enhanced CT, or Doppler ultrasonography. A detailed summary of HHT patient genetics, clinical manifestations, and demographics, as well as control demographics can be seen in Table 1.

### 3.2. HHT MACs Have a Dysregulated MiRNA Profile Enriched in Pathways Involved in HHT Pathogenesis

**Figure 1:**
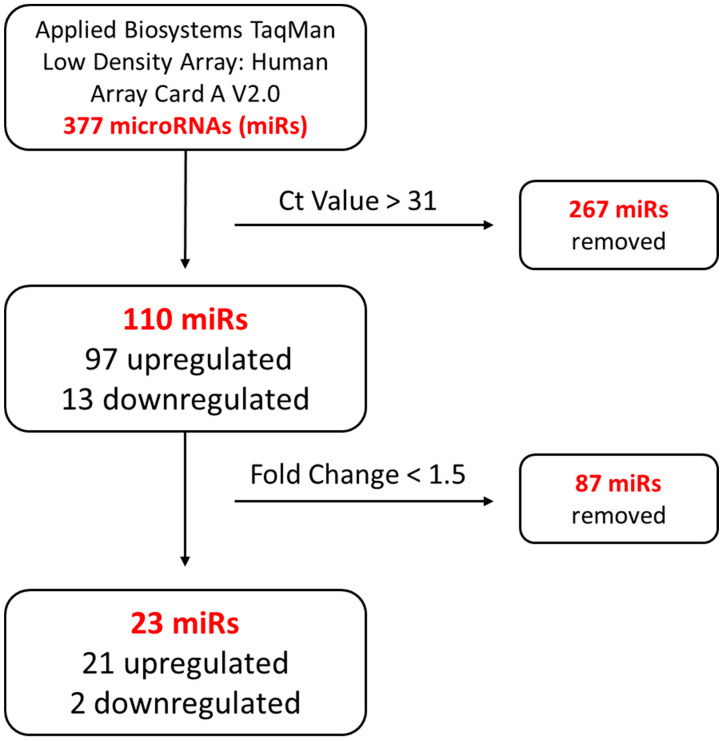
Systematic miRNA exclusion flow chart of miRNA microarray analysis. Ct: cycle threshold value.

**Figure 2:**
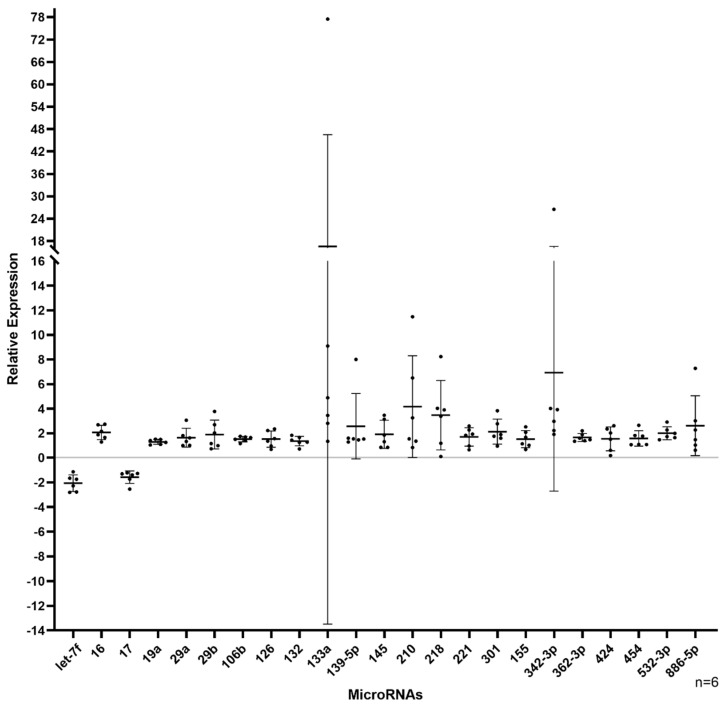
Dot plot of the 23 dysregulated MAC miRNAs. Twenty-one miRNAs were found to be upregulated and two downregulated by the microarray analysis after systematic exclusion.

**Figure 3:**
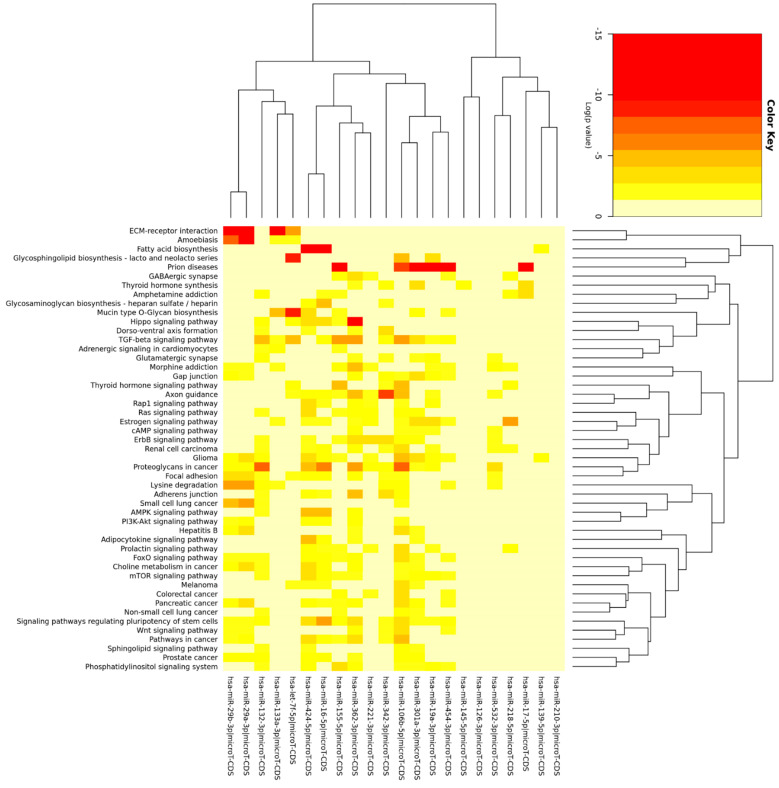
Functional enrichment analysis heat map with hierarchical clustering of the significantly enriched KEGG pathways. The coloured scale bar represents LOG (*p*-value) from 0 to –15 where red indicates more significance and yellow indicates less significance. The background colour of the heat map or 0 of the scale bars represent no significance. Each row represents a KEGG pathway, and each column represents a miRNA. The miRNA clustering tree or dendrogram is shown at the top and the KEGG pathway clustering tree or dendrogram is shown on the right.

**Figure 4:**
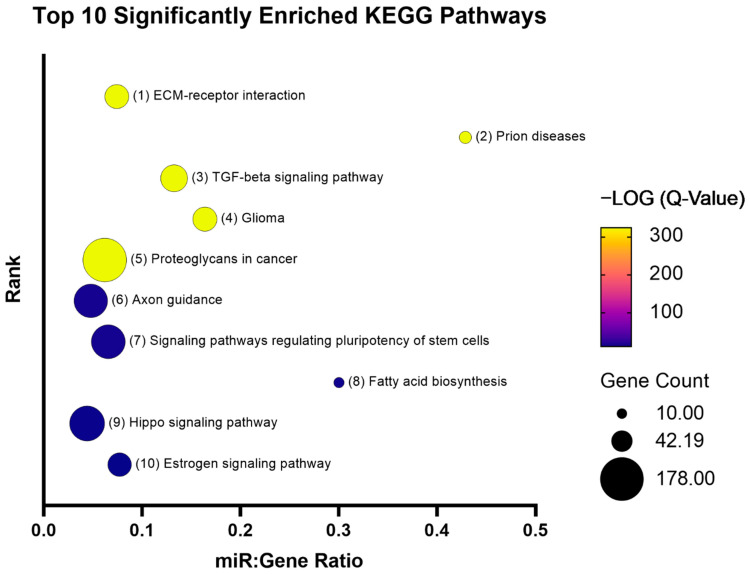
Bubble plot represents the top 10 significantly enriched KEGG pathways for the 23 dysregulated miRNAs. The coloured scale bar indicates the −LOG (Q-value) where yellow indicates more significance and blue indicates less significance. Q-value is the FDR adjusted *p*-value. Bubble size represents the number of genes targeted in that particular pathway (gene count). The *y*-axis represents the pathway rank from most to least significant (1 through 10). The *x*-axis represents the number of miRNAs that are enriched in a particular pathway relative to the number of target genes that are enriched in the same pathway, known as the miR:gene ratio.

MAC characterization results are in line with those that we reported previously [47,48]. TaqMan microarray results were processed as follows, and miRNAs that had a Ct value greater than 31 and a fold change less than 1.5 were systematically removed (Figure 1). A total of 23 miRNAs were identified to be dysregulated (21 upregulated and 2 downregulated) in HHT MACs (Figure 2). A list of the dysregulated miRNAs is shown in Table 2. Functional enrichment analysis in KEGG pathways by DIANA-miRPath v.3 revealed that the dysregulated miRNAs were significantly enriched in pathways related to HHT pathogenesis, such as TGFβ, PI3K/AKT/Ras/MAPK [55,56,57,58,59,60], mTOR [61], Hippo [62], and Wnt [63,64] signalling pathways (Figure 3). The top 10 significantly enriched pathways can be seen in Figure 4. The extracellular matrix (ECM)–receptor interaction pathway (Pathway ID: map04512, FDR adjusted *p* < 1 × 10^−325^) was the most significantly enriched and targeted by miRs-29a/b-3p, -133a-3p, and let-7f-5p. The TGFβ signalling pathway (Pathway ID: map04350, FDR adjusted *p* < 1 × 10^−325^) was the third most significantly enriched, targeted by nine of the dysregulated miRNAs, including miRs-132-3p, -155-5p, -362-3p, -106b-5p, and let-7f-5p, to name a few. A total of 68 genes were targeted in the TGFβ signalling pathway, including *SMAD2*, *SMAD4*, *ID1*, *TGFBR1/2*, and *BMPR2*. A complete list of the significantly enriched KEGG pathways is demonstrated in Table S1.

### 3.3. MiR-132-3p Is Significantly Decreased in HHT MACs

**Figure 5:**
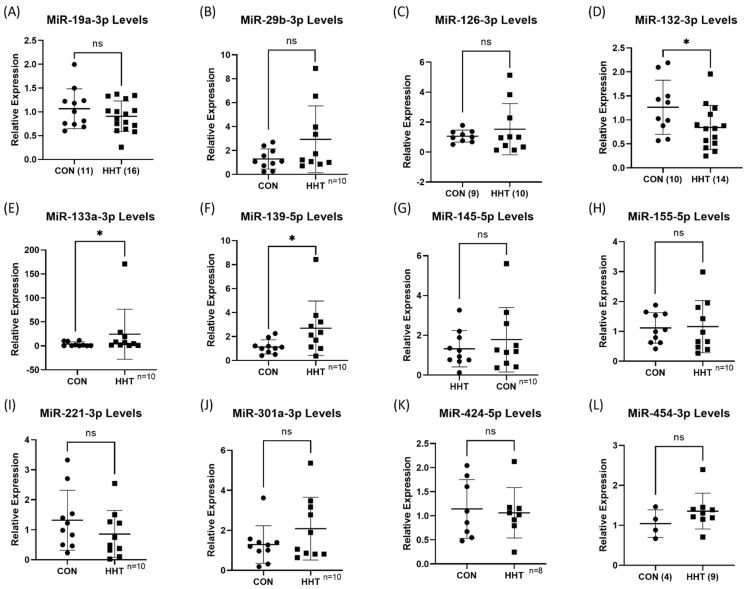
(**A**–**L**) RT-qPCR validation of 12 miRNAs. Three miRNAs as indicated in the graphs were found to be significantly different in HHT and control MACs, while nine as indicated were not. * *p* < 0.05, and ns indicates not statistically significant.

**Figure 6:**
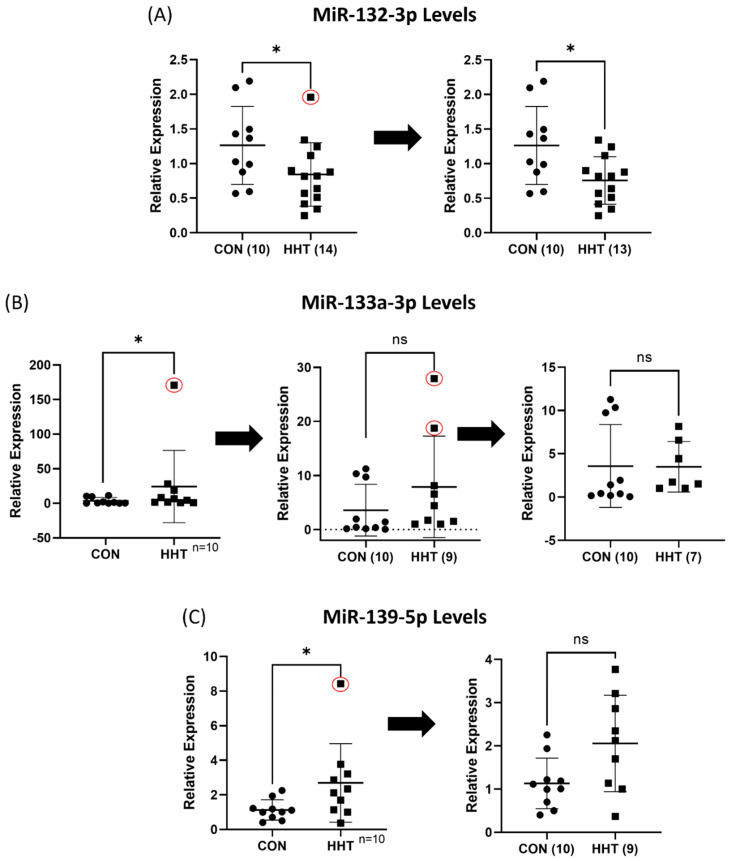
Z-score analysis of outliers. Removal of outliers (circled in red) by Z-score analysis did not result in loss of statistical significance in miR-132-3p (**A**) but in miR-133a-3p (**B**) and miR-139-5p (**C**). * *p* < 0.05 and ns indicates not statistically significant.

Of the 23 dysregulated miRNAs identified by the microarray analysis, 12 miRNAs (miRs-19a-3p, -29b-3p, 126-3p, -132-3p, -133a-3p, -139-5p, -145-5p, -155-5p, -221-3p, -301a-3p, -424-5p, and -454-3p) were selected for RT-qPCR validation. These miRNAs were chosen because they are well characterized, and some have been validated in healthy MACs in a previous study [48]. Levels of miRs-19a-3p, -29b-3p, -126-3p, -145-5p, -155-5p, -221-3p, -301a-3p, -424-5p, and -454-3p were not found to be significantly different between HHT and control MACs by RT-qPCR (Figure 5). MiR-132-3p was shown to be significantly decreased in HHT MACs compared with control MACs. MiRs-139-5p and -133a-3p were found to be significantly increased in HHT MACs (Figure 5). However, the presence of extreme data points or outliers in the HHT MACs were of concern. A Z-score analysis was applied to all the miRNAs measured by RT-qPCR to identify and remove outliers (Figure S1). The removal of outliers for miRs-139-5p and -133a-3p resulted in a loss of significance between HHT and control groups, while miR-132-3p levels remained significantly decreased in HHT MACs (Figure 6). Clinical characteristics, age, gender, mutated genes, or AVM types for miRNA expression outliers are shown in Table S2. No correlations were identified between levels of miR-132-3p and subject age or gender in both groups.

### 3.4. MiR-132-3p Is Significantly Enriched in the TFGβ and PI3K/AKT Signalling Pathways

**Figure 7:**
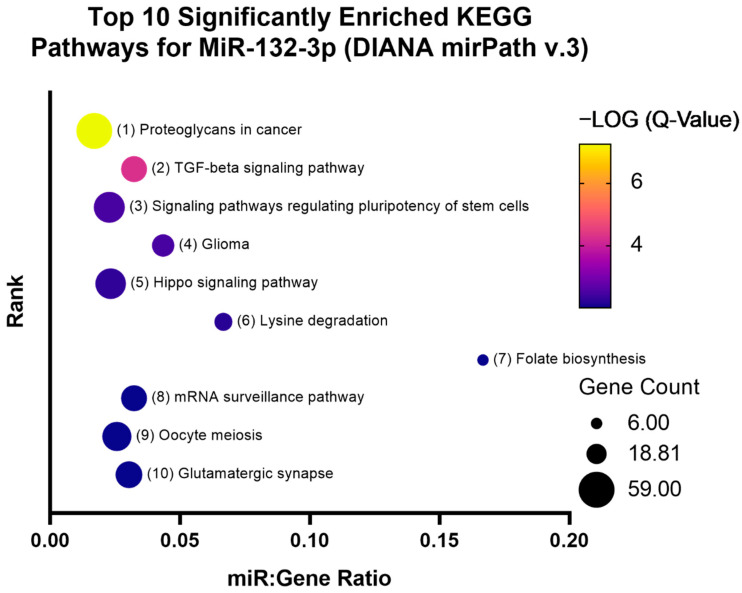
Bubble plot represents the top 10 significantly enriched KEGG pathways for miR-132-3p. The coloured scale bar indicates the −LOG (Q-value) where yellow indicates more significance and blue indicates less significance. Q-value is the FDR adjusted *p*-value. Bubble size represents the number of genes targeted in that particular pathway (gene count). The *y*-axis represents the pathway rank from most to least significant (1 through 10). The *x*-axis represents the number of miRNAs that are enriched in a particular pathway relative to the number of target genes that are enriched in the same pathway, known as the miR:gene ratio.

**Figure 8:**
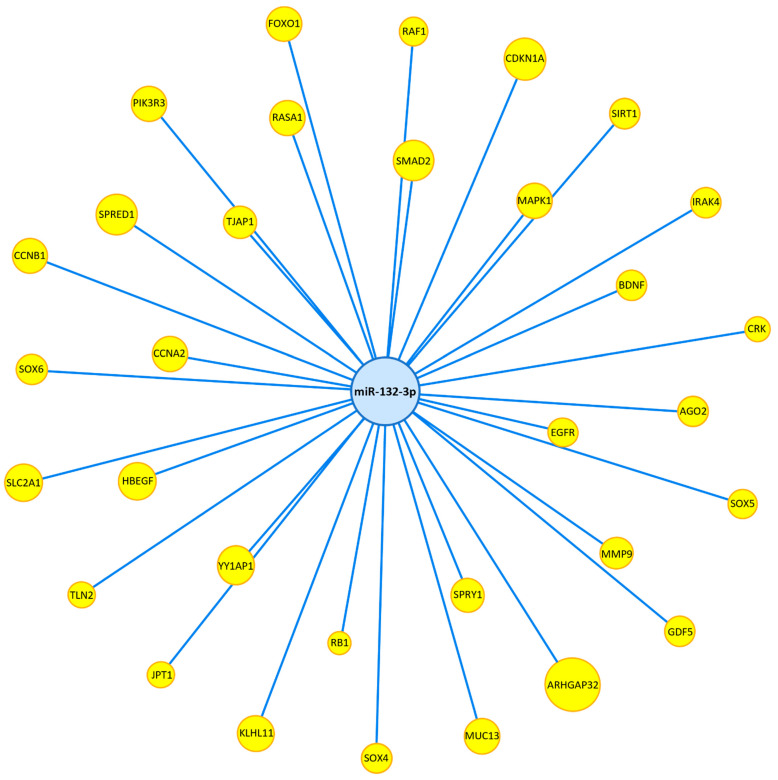
MiR-132-3p target interaction network. All miR-132-3p targets are experimentally validated (Western blot and luciferase assay) as curated by miRTarbase. Yellow nodes represent miR-132-3p target genes. Edges indicate gene–miRNA interactions. Nodes are sized appropriately to fit gene/miRNA names.

**Figure 9:**
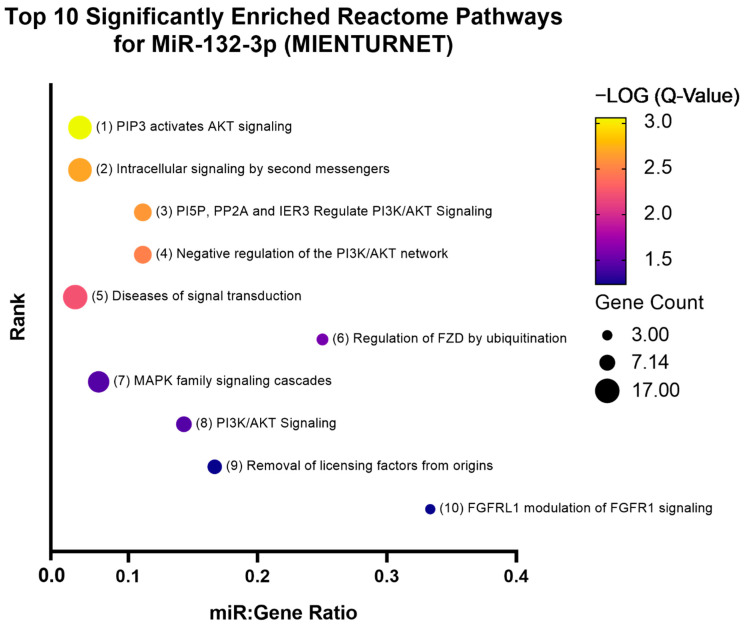
Bubble plot of the top 10 significantly enriched Reactome pathways for miR-132-3p targets identified by MIENTURNET and miRTarbase. The coloured scale bar indicates the −LOG (Q-value) where yellow indicates more significance and blue indicates less significance. Q-value is the FDR adjusted *p*-value. Bubble size represents the number of genes targeted in that particular pathway (gene count). The *y*-axis represents the pathway rank from most to least significant (1 through 10). The *x*-axis represents the number of miRNAs that are enriched in a particular pathway relative to the number of target genes that are enriched in the same pathway, known as the miR:gene ratio.

MiR-132-3p functional enrichment analysis in KEGG pathways by DIANA-miRPath v.3 identified TGFβ signalling as the second most significantly enriched pathway (FDR adjusted *p* = 5.34 × 10^−5^) (Figure 7). A total of 31 genes in the TGFβ signalling pathway were predicted to be targeted by miR-132-3p, including *SMAD2*, *SMAD4*, *GDF5*, and *BMPR2*. A full list of predicted miR-132-3p target genes in the TGFβ signalling pathway can be seen in Table 3. Target enrichment analysis by MIENTURNET using the experimentally validated miRNA–target interactions database, miRTarbase v9.0, identified 33 experimentally validated (Western blot or luciferase assay) targets of miR-132-3p, including, *RASA1*, *SMAD2*, and *GDF5* (Figure 8). Functional enrichment analysis of these targets in Reactome pathways by MIENTURNET identified regulatory pathways of PI3K/AKT signalling as the most significantly enriched, including the Reactome pathways, “PIP3 activates AKT signaling” (Pathway ID: R-HSA-1257604, FDR adjusted *p* = 0.0008), “PI5P, PP2A, and IER3 regulate PI3K/AKT signaling” (Pathway ID: R-HSA-6811558, FDR adjusted *p* = 0.0024), and “negative regulation of PI3K/AKT network” (Pathway ID: R-HSA-199418, FDR adjusted *p* = 0.0032) (Figure 9). A complete list of enriched Reactome pathways and their respective miR-132-3p target genes can be seen in Table S3.

## 4. Discussion

In the present study, we demonstrated that HHT MACs have a dysregulated miRNA profile with a significant reduction in miR-132-3p expression. Of note, miR-132-3p levels in some healthy controls were found to be as low as those in patients. The reason for this remains unresolved. Lifestyle factors such as diet, physical activity, and alcohol consumption, known to affect miRNA expression [65], might be responsible for miR-132-3p variation in healthy controls. Bioinformatic analysis revealed that miR-132-3p is significantly enriched in the TGFβ and PI3K/AKT signalling pathways, both known to be involved in HHT pathogenesis [55,56,57]. MiR-132-3p is well characterized in various neurological and oncological pathologies, including Alzheimer’s and Parkinson’s disease, as well as lung, breast, glioma, and cervical cancer, to name a few [66,67]. MiR-132-3p has been shown to be a critical regulator of cellular apoptosis, migration, adhesion, and proliferation through various pathways, including the TGFβ, PI3K/AKT/Ras/MAPK, mTOR, Hippo, and Hedgehog signalling pathways [67].

MiR-132-3p has been shown to be induced by TGFβ1/2 ligands and is in fact directly controlled by the TGFβ signalling pathway [68,69,70]. It is not surprising that HHT MACs, which presumably have downregulated TGFβ signalling due to the genetic mutations, have reduced expression of miR-132-3p. Wang et al. demonstrated that miR-132-3p expression is controlled by TGFβ in a time- and concentration-dependent manner in glioma cells [69]. They also found miR-132-3p could enhance TGFβ signalling by directly targeting *SMAD7*, a negative regulator of TGFβ signalling [69]. Similarly, Li et al. showed that miR-132-3p expression could be stimulated via the induction of TGFβ1/2 in keratinocytes [70]. They also showed that inhibition of miR-132-3p could delay skin wound healing, increase inflammation, and reduce keratinocyte proliferation in a mouse model [70]. Li et al. further demonstrated that miR-132-3p expression can be induced by TGFβ1 in human dermal fibroblasts (HDFs) [68]. They also showed that miR-132-3p inhibition can delay skin wound healing in a human surgical wound model [68]. Interestingly, they demonstrated that miR-132-3p inhibition can impair HDF migration by directly targeting RASA1 [68]. RASA1 is a negative regulator of the PI3K/AKT and Ras/MAPK signalling pathways and plays a pivotal role in the regulation of cellular proliferation, apoptosis, and migration [71]. These data indicate that miR-132-3p promotes cell migration and proliferation in various cell types. In view of this, we speculate that the previously reported impairment of HHT MAC migration and proliferation might be due in part to reduced levels of miR-132-3p. Whether overexpression of miR-132-3p would rescue HHT MAC function warrants further study. As miR-132-3p targets RASA1, a negative regulator of key pathways, such as the PI3K/AKT pathway, that stimulate cell migration and growth, it would be of interest to examine RASA1 and its target pathway expression in HHT MACs to better understand the contribution of miR-132-3p to HHT MAC dysfunction.

Several studies have also identified that miR-132-3p regulates EC function and angiogenesis by targeting RASA1. Anand et al. revealed that miR-132-3p overexpression in HUVECs increased cell proliferation and tube forming capacity, while inhibition of miR-132-3p in the mouse postnatal retina impaired vascular development [72]. They further determined that miR-132-3p exerts its effects through RASA1, where the delivery of anti-miR-132-3p to vessel endothelium in an orthotopic xenograft mouse model of breast carcinoma restored RASA1 expression and suppressed angiogenesis [72]. The proangiogenic effects of miR-132-3p were also demonstrated by Devalliere et al., where HUVECs transfected with miR-132-3p had enhanced proliferation, migration, and vascularization [73]. Lei et al. demonstrated that the overexpression of miRs-132-3p and -212 in HUVECs enhanced vascularization through the direct inhibition of RASA1 and SPRED1 [74]. Interestingly, mesenchymal stromal cell-derived exosomes (MSC-EXs) loaded with miR-132-3p have been shown to promote EC function through the inhibition of RASA1. Ma et al. demonstrated that MSC-EXs containing miR-132-3p increased HUVEC tube formation and angiogenic capacity by targeting RASA1 [75]. Pan et al. showed that MSC-EXs loaded with miR-132-3p decreased reactive oxygen species production, apoptosis, and tight junction disruption in hypoxia/reoxygenation injured mouse brain microvascular ECs compared with MSC-EXs without miR-132-3p [76]. These effects were due to increased PI3K/AKT and Ras/MAPK signalling as a result of miR-132-3p inhibiting RASA1 and associated with increased expression of eNOS and phospho-AKT [76]. Taken together, miR-132-3p can promote cell proliferation and migration and inhibit apoptosis in a variety of cell types by suppressing RASA1, a negative regulator of the PI3K/AKT and Ras/MAPK signalling pathways. Therefore, it is possible that reduced levels of miR-132-3p may be contributing to the observed HHT MAC dysfunction.

Differences in disease expression only partially reflect the specific gene that is mutated in HHT. Even within families with the same genetic mutation, expressivity can widely vary. We found in the present study that miR-132-3p is enriched in signalling pathways that are involved in HHT pathogenesis. It is possible that the degree of downregulation of miR-132-3p might better correlate with phenotype expression, although we did not follow our patients longitudinally. In this sense, measurement of MAC miR-132-3p might predict individuals who are at higher risk of AVM formation and bleeding, and allow for tailored pharmacotherapy and clinical follow-up. Longitudinal clinical studies are necessary to test the hypothesis.

In conclusion, miRNA dysregulation, specifically reduced expression of miR-132-3p, in HHT MACs was identified. The dysregulated miRNAs were significantly enriched in the TGFβ, PI3K/AKT, and Hippo signalling pathways. These data suggest that miRNA alteration may impair these pathways, resulting in MAC dysfunction, which warrants further study.

## Figures and Tables

**Table 1:** Summary of demographics and clinical information.

	HHT Patients (*n* = 40)	Controls (*n* = 22)
Age (years)	49.4 ± 10.6	46.2 ± 12.6
Number (%) of Females	21 (52.5)	12 (54.5)
Average Female Age (years) (SD)	50.0 ± 9.8	48 ± 11.2
Number (%) of Males	19 (47.5)	10 (45.5)
Average Male Age (years) (SD)	48.8 ± 11.7	44 ± 14.5
**Mutated Genes (Number of HHT Patients):**		
*ENG*	19 (47.5)	
*ACVRL1*	15 (37.5)	
*SMAD4*	1 (2.5)	
Unknown *	5 (12.5)	
**AVMs (Number of HHT Patients):**		
PAVM	17 (42.5)	
PAVM and CAVM	3 (7.5)	
PAVM and LVM	2 (5)	
CAVM LVM	1 (2.5) 3 (7.5)	
No AVM Unknown †	13 (32.5) 1 (2.5)	

Data presented as mean ± standard deviation (SD) or *n* (%). * Patients without an identified mutation despite full mutational analysis in the known HHT genes, including *ENG*, *ACVRL1*, and *SMAD4*, or patients who have not yet undergone genetic testing. † The patient had not undergone AVM screening. Abbreviations: HHT, hereditary hemorrhagic telangiectasia; *ENG*, endoglin; *ACVRL1*, activin receptor-like kinase 1; *SMAD4*, mothers against decapentaplegic homolog 4; AVM, arteriovenous malformation; PAVM, pulmonary arteriovenous malformation; CAVM, cerebral arteriovenous malformation; LVM, liver (hepatic) vascular malformation.

**Table 2:** Dysregulated miRNAs identified by the microarray analysis. Table includes the miRNA accession code (miRBase Identification Code), microarray assay target sequence, and fold change.

MicroRNAs	Accession	Assay Target Sequence	Fold Increase
hsa-miR-16-5p	MIMAT0000069	UAGCAGCACGUAAAUAUUGGCG	2.06
hsa-miR-19a-3p	MIMAT0000073	UGUGCAAAUCUAUGCAAAACUGA	1.50
hsa-miR-29a-3p	MIMAT0000086	UAGCACCAUCUGAAAUCGGUUA	1.63
hsa-miR-29b-3p	MIMAT0000100	UAGCACCAUUUGAAAUCAGUGUU	1.89
hsa-miR-106b-5p	MIMAT0000680	UAAAGUGCUGACAGUGCAGAU	1.52
hsa-miR-126-3p	MIMAT0000445	UCGUACCGUGAGUAAUAAUGCG	1.69
hsa-miR-132-3p	MIMAT0000426	UAACAGUCUACAGCCAUGGUCG	1.54
hsa-miR-133a-3p	MIMAT0000427	UUUGGUCCCCUUCAACCAGCUG	16.51
hsa-miR-139-5p	MIMAT0000250	UCUACAGUGCACGUGUCUCCAG	2.56
hsa-miR-145-5p	MIMAT0000437	GUCCAGUUUUCCCAGGAAUCCCU	1.91
hsa-miR-210-3p	MIMAT0000267	CUGUGCGUGUGACAGCGGCUGA	4.16
hsa-miR-218-5p	MIMAT0000275	UUGUGCUUGAUCUAACCAUGU	3.46
hsa-miR-221-3p	MIMAT0000278	AGCUACAUUGUCUGCUGGGUUUC	1.70
hsa-miR-301a-3p	MIMAT0000688	CAGUGCAAUAGUAUUGUCAAAGC	2.13
hsa-miR-155-5p	MIMAT0000646	UUAAUGCUAAUCGUGAUAGGGGU	1.68
hsa-miR-342-3p	MIMAT0000753	UCUCACACAGAAAUCGCACCCGU	6.92
hsa-miR-362-3p	MIMAT0004683	AACACACCUAUUCAAGGAUUCA	1.64
hsa-miR-424-5p	MIMAT0001341	CAGCAGCAAUUCAUGUUUUGAA	1.82
hsa-miR-454-3p	MIMAT0003885	UAGUGCAAUAUUGCUUAUAGGGU	1.68
hsa-miR-532-3p	MIMAT0004780	CCUCCCACACCCAAGGCUUGCA	2.00
hsa-miR-886-5p	MI0005527	CGGGUCGGAGUUAGCUCAAGCGG	2.61
			**Fold Decrease**
hsa-let-7f-5p	MIMAT0000067	UGAGGUAGUAGAUUGUAUAGUU	2.07
hsa-miR-17-5p	MIMAT0000070	CAAAGUGCUUACAGUGCAGGUAG	1.59

**Table 3:** List of miR-132-3p target genes in the TGFβ signalling pathway returned from DIANA miRPath v.3. MicroT-CDS score is the miRNA target prediction algorithm score where values closer to 1 are highly predicted. Experimental support refers to whether the predicted target has been supported by experimental evidence, such as Western blot or luciferase assay.

Gene Name	Gene Ensembl ID	Microt-CDS Score	Experimentally Supported
*ROCK1*	ENSG00000067900	0.921	No
*SMAD2*	ENSG00000175387	0.937	Yes
*INHBB*	ENSG00000163083	0.564	No
*SMAD9*	ENSG00000120693	0.871	No
*THBS1*	ENSG00000137801	0.606	Yes
*BMP5*	ENSG00000112175	0.737	No
*CDKN2B*	ENSG00000147883	0.684	No
*ACVR1*	ENSG00000115170	0.914	No
*SKP1*	ENSG00000113558	0.669	No
*ACVR2B*	ENSG00000114739	0.999	Yes
*ZFYVE16*	ENSG00000039319	0.531	No
*DCN*	ENSG00000011465	0.517	No
*SMAD4*	ENSG00000141646	0.711	No
*E2F5*	ENSG00000133740	0.503	Yes
*SMURF1*	ENSG00000198742	0.691	Yes
*ZFYVE9*	ENSG00000157077	0.716	No
*SMAD5*	ENSG00000113658	0.943	Yes
*GDF6*	ENSG00000156466	0.527	No
*MAPK3*	ENSG00000102882	0.608	No
*TFDP1*	ENSG00000198176	0.854	No
*SP1*	ENSG00000185591	0.538	Yes
*GDF5*	ENSG00000125965	0.998	No
*TGFB2*	ENSG00000092969	0.855	No
*EP300*	ENSG00000100393	1.000	No
*PPP2CB*	ENSG00000104695	0.800	Yes
*BMPR1A*	ENSG00000107779	0.605	No
*LTBP1*	ENSG00000049323	0.611	No
*MAPK1*	ENSG00000100030	0.992	Yes
*PPP2R1B*	ENSG00000137713	0.587	Yes
*BMPR2*	ENSG00000204217	0.717	No
*RPS6KB1*	ENSG00000108443	0.515	No

## Data Availability

All data are included in the article and Supplementary Materials.

## Supplementary Materials

The following supporting information can be downloaded at: https://www.mdpi.com/article/10.3390/genes13040665/s1, Figure S1: RT-qPCR validation of the miRs identified by microarray analysis after outlier removal by Z-score analysis; Table S1: Significantly enriched KEGG pathways returned from DIANA-miRPath v.3 analysis. Q-value is the FDR adjusted P-value. MiR:gene ratio is the number of miRs that are enriched in a particular pathway relative to the number of target genes that are enriched in the same pathway; Table S2: Clinical characteristics of HHT patient outliers identified by Z-Score analysis; Table S3: List of the top 10 enriched Reactome pathways returned from the functional enrichment analysis of miR-132-3p targets. Q-value is the FDR adjusted P-value. MiR:gene ratio is the number of miRs that are enriched in a particular pathway relative to the number of target genes that are enriched in the same pathway.
