# Supplementary material for: MicroRNA-132-3p, Downregulated in Myeloid Angiogenic Cells from Hereditary Hemorrhagic Telangiectasia Patients, Is Enriched in the TGFβ and PI3K/AKT Signalling Pathways
Source: Genes (Basel). 2022 Apr 9;13(4):665. doi: 10.3390/genes13040665 (PMC9027908; doi:10.3390/genes13040665)

Supplementary Materials:

Table S1. Significantly enriched KEGG pathways returned from DIANA-miRPath v.3 analysis. Q-value is the FDR adjusted P-value. MiR:gene ratio is the number of miRs that are enriched in a particular pathway relative to the number of target genes that are enriched in the same pathway.

KEGG Pathway	Q-Value (FDR Adjusted P Value)	-LOG10 (Q-Value)	Number of Genes	Number of MiRs	miR:Gene Ratio
ECM-receptor interaction	<1E-325	>325	54	4	0.074074
Prion diseases	<1E-325	>325	14	6	0.428571
TGF-beta signaling pathway	<1E-325	>325	68	9	0.132353
Glioma	<1E-325	>325	55	9	0.163636
Proteoglycans in cancer	<1E-325	>325	178	11	0.061798
Axon guidance	3.33E-16	15.47756	105	5	0.047619
Signaling pathways regulating pluripotency of stem cells	9.99E-16	15.00043	107	7	0.065421
Fatty acid biosynthesis	2.42E-14	13.61618	10	3	0.3
Hippo signaling pathway	2.16E-12	11.66555	114	5	0.04386
Estrogen signaling pathway	8.25E-12	11.08355	52	4	0.076923
Lysine degradation	6.66E-11	10.17653	30	5	0.166667
ErbB signaling pathway	1.79E-10	9.747147	65	5	0.076923
Pancreatic cancer	3.73E-10	9.428291	56	6	0.107143
Pathways in cancer	3.85E-10	9.414539	306	5	0.01634
Focal adhesion	4.29E-10	9.367543	149	5	0.033557
FoxO signaling pathway	1.10E-09	8.958607	100	4	0.04
Prostate cancer	2.93E-09	8.533132	69	7	0.101449
Mucin type O-Glycan biosynthesis	3.18E-09	8.497573	24	4	0.166667
Choline metabolism in cancer	4.51E-09	8.345823	78	5	0.064103
Phosphatidylinositol signaling system	2.79E-08	7.554396	64	4	0.0625
mTOR signaling pathway	6.36E-08	7.196543	48	4	0.083333
Prolactin signaling pathway	1.54E-07	6.812479	46	3	0.065217
Thyroid hormone signaling pathway	2.83E-07	6.548214	75	4	0.053333
Renal cell carcinoma	9.05E-07	6.043351	55	3	0.054545
AMPK signaling pathway	1.62E-06	5.790485	85	3	0.035294
Ras signaling pathway	2.14E-06	5.669586	169	5	0.029586
Morphine addiction	3.23E-06	5.490797	59	3	0.050847
Amoebiasis	6.59E-06	5.181115	39	2	0.051282
Wnt signaling pathway	6.87E-06	5.163043	79	2	0.025316
Glycosphingolipid biosynthesis - lacto and neolacto series	1.06E-05	4.974694	17	3	0.176471
Hepatitis B	1.71E-05	4.767004	73	3	0.041096
Melanoma	3.03E-05	4.518557	34	1	0.029412
PI3K-Akt signaling pathway	3.35E-05	4.474955	227	4	0.017621
Gap junction	3.43E-05	4.464706	59	5	0.084746
Rap1 signaling pathway	3.83E-05	4.416801	158	3	0.018987

Small cell lung cancer	0.000102	3.99091	37	2	0.054054
Sphingolipid signaling pathway	0.000527	3.278091	48	1	0.020833
Adherens junction	0.000593	3.226786	63	4	0.063492
Thyroid hormone synthesis	0.000725	3.139491	31	4	0.129032
Glycosaminoglycan biosynthesis - heparan sulfate / heparin	0.000859	3.065991	14	2	0.142857
cAMP signaling pathway	0.000937	3.028148	76	1	0.013158

Table S2. Clinical characteristics of HHT patient outliers identified by Z-Score analysis.

HHT Patient ID	MicroRNAs (Z-Score)	Age	Gender	Mutation	AVM
HHT 39	424-5p (2.13)	58	F	*ACVRL1*	PAVM (treated)
HHT 40	133a-3p (2.13)	61	M	*ENG*	None
HHT 57	132-3p (2.43), 454-5p (2.32)	56	F	*ENG*	CAVM (resected)
HHT 67	133a-3p (2.80), 139-5p (2.52)	58	M	*ENG*	PAVM (treated) & CAVM (untreated)
HHT 84	19a-3p (-2.02)	60	M	*ACVRL1*	None
HHT 90	221-3p (2.15)	58	F	*ENG*	PAVM (treated)
HHT 98	29b-3p (2.16), 126-3p (2.11), 145-5p (2.37), 301a-3p (2.08)	43	F	*ENG*	PAVM (treated)
HHT 99	29b-3p (2.12), 126-3p (2.23), 155-5p (2.10)	46	M	*ACVRL1*	None

Table S3. List of the top 10 enriched Reactome pathways returned from the functional enrichment analysis of miR-132-3p targets. Q-value is the FDR adjusted P-value. MiR:gene ratio is the number of miRs that are enriched in a particular pathway relative to the number of target genes that are enriched in the same pathway.

Reactome Pathway	Q-Value (FDR Adjusted P-Value)	-LOG (Q-Value)	Number of Genes	miR:Gene Ratio	Target Genes
PIP3 activates AKT signaling	0.000867	3.062036	16	0.0625	CDKN1A/HBEGF/PIP5K1A/PSMD12/FGF22/PSMA2/FOXO1/IRAK1/MAPK1/TNRC6C/IRAK4/EGFR/FGF2/AGO2/PIK3R3/OTUD3
Intracellular signaling by second messengers	0.002103	2.677078	16	0.0625	CDKN1A/HBEGF/PIP5K1A/PSMD12/FGF22/PSMA2/FOXO1/IRAK1/MAPK1/TNRC6C/IRAK4/EGFR/FGF2/AGO2/PIK3R3/OTUD3
PI5P, PP2A and IER3 Regulate PI3K/AKT Signaling	0.002446	2.611611	9	0.111111	HBEGF/PIP5K1A/FGF22/IRAK1/MAPK1/IRAK4/EGFR/FGF2/PIK3R3
Negative regulation of the PI3K/AKT network	0.003295	2.482164	9	0.111111	HBEGF/PIP5K1A/FGF22/IRAK1/MAPK1/IRAK4/EGFR/FGF2/PIK3R3

Diseases of signal transduction	0.006025	2.220039	17	0.058824	CDKN1A/HBEGF/RASA1/WNT3A/PSMD12/FGF22/PSMA2/SPRED1/FOXO1/MAPK1/FXR1/FZD6/EGFR/RAF1/FGF2/PIK3R3/SMAD2
Regulation of FZD by ubiquitination	0.02717	1.565911	4	0.25	WNT3A/LGR4/FZD6/USP8
MAPK family signaling cascades	0.035587	1.448704	13	0.076923	HBEGF/RASA1/PSMD12/FGF22/PSMA2/SPRED1/FOXO1/MAPK1/TNRC6C/EGFR/RAF1/FGF2/AGO2
PI3K/AKT Signaling	0.035587	1.448704	7	0.142857	CDKN1A/HBEGF/FGF22/FOXO1/EGFR/FGF2/PIK3R3
Removal of licensing factors from origins	0.057979	1.236727	6	0.166667	CDKN1A/RB1/PSMD12/PSMA2/CCNA2/GMNN
FGFRL1 modulation of FGFR1 signaling	0.058074	1.236018	3	0.333333	FGF22/SPRED1/FGF2

Figure S1. RT-qPCR validation of the miRs identified by microarray analysis after outlier removal by Z-score analysis.

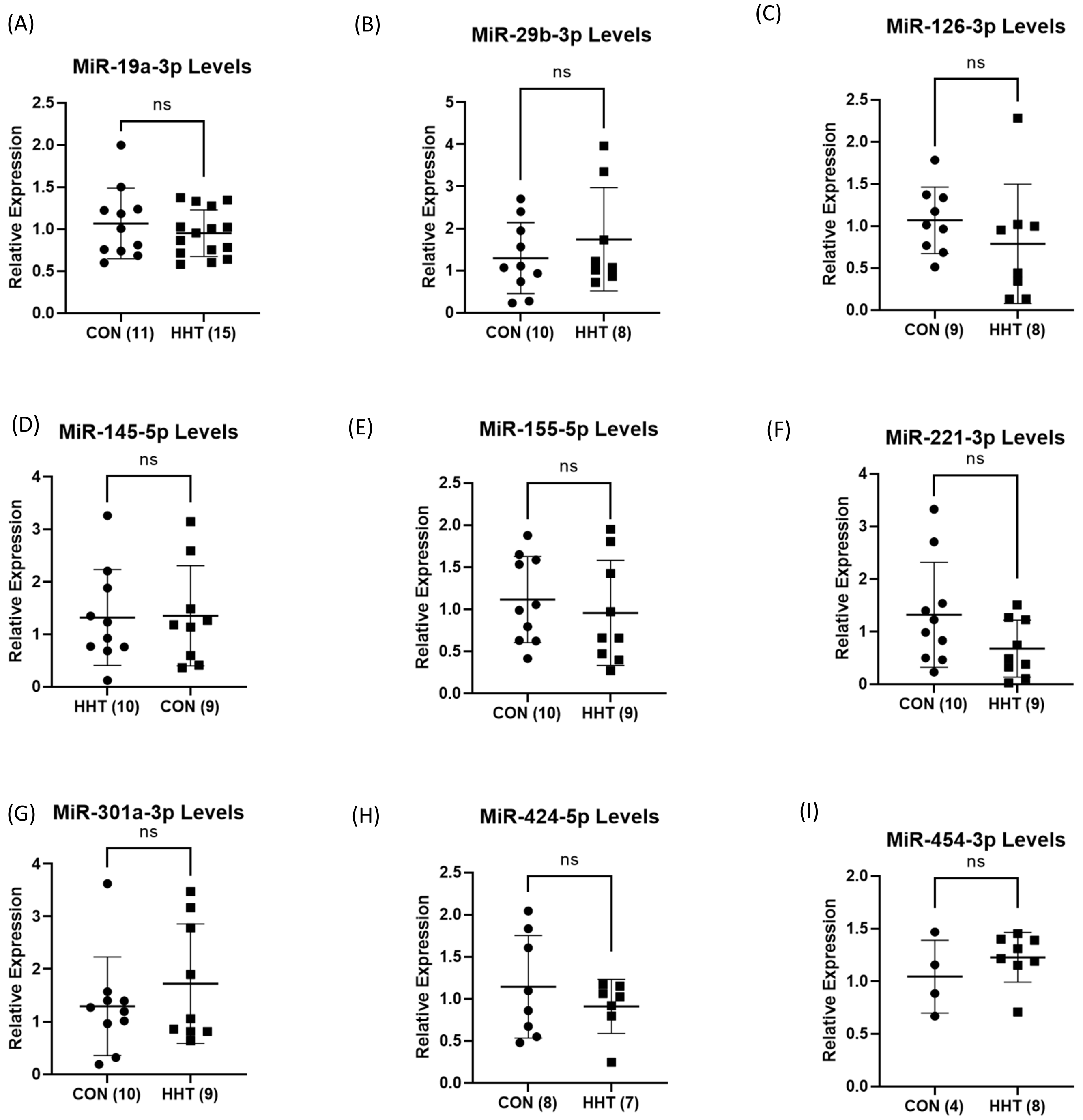

Supplement: Supplementary file 1 [file genes-13-00665-s001.zip › genes-1639850-supplementary.pdf]
